# Abstract of the Proceedings of the Buffalo Medical Association

**Published:** 1855-10

**Authors:** Sanford B. Hunt


ART. IV.—*Abstract of the Proceedings of the Buffalo Medical Association.*

TUESDAY EVENING, Sept. 4, 1855

Association met at the new room which, since the last meeting, had been neatly and conveniently fitted up for its use, under the direction of the committee on rooms and fixtures.

Present—Drs. White, Wyckoff, Wilcox, Flint, Burwell, Gay, Hutchins, Baker, Newman, Whitney, Root, Howell, Lay, Nelson, Nott, Johnson, Hubbard, Hunt, Eastman, Rochester and Gould.

Both President and Vice-president being absent, on motion of Dr. Root, Dr. White was appointed President *pro. tem.*, and presided during the evening.

The minutes of the preceding meeting were read and approved.

On motion of Dr. Burwell, Dr. Julius F. Miner was elected a member of the association.

Dr. Newman then read the report of the committee on rooms (Drs. Newman, Wilcox, and Wyckoff,) giving a statement of their expenditures, and congratulating the association on the possession of a permanent place of meeting.

The report was accepted, and the committee continued with the thanks of the association.

*Dr. Flint*, from the committee on pneumonia, stated that the committee had agreed to present separate reports, inasmuch as they found it impracticable to find time to hold the necessary meetings and discussions incident to the preparation of a unanimous report. Inasmuch as his own paper was much the longest, it had also been agreed that Drs. Gay and Burwell should present theirs first, leaving his to follow them.

*Dr. Gay* then read the following:

*Committee on Pneumonia.* Report of Dr. C. C. F. Gay.

*Twenty-six cases* of pneumonia were treated during the spring of 1853, extending over a period of two and a half months.

10 cases were bled within the first 48 hours.
4 " " on 3d day.
3 " " on 4th "
1 " " on 5th "
8 " were not bled.

Having arranged them in a tabular form I herewith present them to the society.

V. S. FIRST FORTY-EIGHT HOURS.

| Age. | Sex. | No. of V. S | Ounces of Blood taken. | Day of Convalescence. | Average Duration. |
|---|---|---|---|---|---|
| 41 | M. | 1 | 32 | 13 | |
| 18 | M. | 1 | 12 | 6 | |
| 15 | F. | 1 | 10 | 8 | |
| 6 | M. | 1 | 8 | 14 | |
| 43 | M. | 1 | 24 | 7 | |
| 45 | M. | 1 | 20 | 9 | 8·2 days. |
| 29 | F. | 1 | 14 | 7 | |
| 28 | M. | 1 | 12 | 6 | |
| 16 | M. | 1 | 8 | 5 | |
| 17 | F. | 1 | 9 | 7 | |

V. S. THIRD DAY.

| Age. | Sex. | No. of V. S. | Ounces of Blood taken. | Day of Convalescence. | Average Duration. |
|---|---|---|---|---|---|
| 14 | M. | 1 | 10 | 7 | |
| 38 | F. | 1 | 16 | 7 | |
| 20 | M. | 1 | 14 | 6 | 7·25 days. |
| 39 | F. | 1 | 16 | 9 | |

V. S. FOURTH DAY.

| Age. | Sex. | No. of V. S | Ounces of Blood taken. | Day of Convalescence. | Average Duration. |
|---|---|---|---|---|---|
| 22 | M. | 1 | 8 | 10 | |
| 23 | F. | 1 | 8 | 20 | 14·33 days. |
| 21 | M. | 1 | 6 | 13 | |

V. S. FIFTH DAY.

| Age. | Sex. | No. of V. S. | Ounces of Blood taken. | Day of Convalescence. | |
|---|---|---|---|---|---|
| 18 | M. | 1 | 3 | 14 | |

There remain eight cases in which V. S. was not practiced. In these the average period of convalescence was thirteen days.

I will briefly detail a few cases, which may serve as a sample of the whole.

1st. V. S. employed the first forty-eight hours.

Mr. B., age 41, full habit; previous health good; had a chill in night, succeeded by fever and pain in side. Saw him a few hours after his attack.

Prescribed V. S., the patient sitting upright in his chair. The abstraction of about four ounces produced syncope; placing him in the recumbent posture I bled to thirty ounces, reducing the pulse from 130 to 90 per minute.

General symptoms, forty-eight hours from attack, same as when first seen with addition of cough and expectoration of blood. V. S. now as apparently indicated as when first seen. Did not yield to patient's importunities to repeat it, but prescribed the ordinary remedies, under which the disease yielded, and the patient was convalescent on thirteenth day.

Fourteen years prior to this time he had an attack of pneumonia, for which he was not bled, and he assured me he was not rid of all traces of the disease for twelve months. From this second attack he was well, in every respect, in four weeks, which he very naturally attributed to blood-letting.

Mr. D., age 43; sanguine temperament, accustomed to active out-door exercise.

General symptoms—Pulse 120, hard; respiration 35; cough, and expectoration of rusty sputa; pain in side; countenance flushed; high degree of fever.

Saw this patient shortly after his attack.

Prescribed V. S., 24 ounces, reducing at once the frequency of pulse and number of respirations. The improvement was only temporary. Exacerbation of all the symptoms, forty-eight hours afterward. Did not repeat the bleeding. Patient convalescent ninth day.

2d. V. S. employed the third day.

Mrs. W., age 39. Had been under Thompsonian treatment, and taken three or four lobelia emetics; had been purged, &c., without any amendment.

General symptoms—Pain in side severe; breathing hurried; cough, bloody expectoration; pulse 98, hard; high degree of fever.

Prescribed V. S. 16 oz., which produced immediate and permanent relief, requiring little or no subsequent treatment.

Mr. L., age 14. As this was the third case treated in the same family, the patient possessing very great repugnance to being bled, I withheld the lancet and otherwise gave him active treatment, in which I persisted until the third day, without any abatement of the inflammation.

General symptoms at this time, substantially same as last case.

Prescribed V. S. 10 oz., the relief being almost instantaneous and permanent.

3d. V. S. employed the fourth day.

Mr. C., Welchman, age 22, laborer; was taken sick with a chill, succeeded by fever and pain in side. Took some pills and other domestic remedies, and tried himself to overcome the disease without calling medical assistance.

General symptoms the fourth day — Pulse 100, full; respiration 35; cough, expectoration occasionally streaked with blood.

Physical signs — Dullness on percussion, over right lung posteriorly, with corresponding auscultatory signs of hepatization.

Prescribed V. S. 8 oz. This amount of depletion was all he would bear.

I am in doubt whether he was in the least benefited or injured by this loss of blood.

The after treatment, which was mild, consisted mainly of tart. ant. et. pot., combined with hydrochlorate of ammonia, as an expectorant; and proto chlo· hydr., combined with pulv. ipecacuanha comp., as an alterative. The patient was convalescent on tenth day.

4th. V. S. employed the fifth day.

Mr. B., age 18. Was called in consultation to see this patient. He had not been bled. Been sick four days, constantly growing worse.

Functional symptoms — Pulse 130; respiration 45; dry cough; had previously expectorated blood.

Physical signs indicative of solidification of right lung.

After much doubt and hesitancy, V. S. was decided upon; after detracting not to exceed three ounces, the pulse began to flag, and the patient to feel faint; obliged to have recourse to ammonia, under the use of which the patient rallied, and was convalescent on fourteenth day.

With one exception, this is the only patient I have seen bled on the fifth day. This was a boy, six years of age, whom I recently visited by invitation of Dr. Burwell. The upper lobe of right lung was solidified; he was bled for the third time, on the fifth day, which was well borne, the little patient feeling less oppression after it, and recovered.

Of my patients treated by depletion, V. S. was not practiced *but once*, and always carried either to complete or incipient syncope.

I am compelled to believe it injudicious to prescribe or proscribe V. S. indiscriminately in every case, or to practice it *too early* as well as *too late.* To derive the fullest advantage from bloodletting, it should be *practiced only as the inflammation approaches its culminating point.*

It will be observed from the table of cases I have collected, that those bled at the beginning of the third day were soonest to recover, the impression being soonest felt.

The same general rule by which we are governed in the application of a blister to the chest may be applied in the use or disuse of V. S., there being a *period* in the *sthenic* form of the disease when it is actually demanded; but if practiced before, or subsequent to, would be of no service, but of positive detriment.

Formerly vesecation was had recourse to in the early stage of the disease; at the present day no physician would think of prescribing it. May we not expect a like reform in the use of the lancet?

Again, it will be observed the mean duration of the disease in the fourteen cases bled within the first three days, was 7·9 days; while the mean duration of the eight cases not bled, was 13 days.

Now when we take into account the fact that the latter were milder forms of disease, it becomes apparent, so far as these cases are of any worth, that venesection abridges the duration of the disease; that convalescence, instead of being retarded, is accelerated, and the proclivity to typhoid symptoms warded off.

I have never supposed pneumonia to be attended with any considerable degree of mortality: on the contrary, believe forty-nine out of fifty cases, exclusive of the extremes of life, will recover if judiciously treated.

To show the influence of bloodletting in pneumonia, Dr. Routh, of London, having given this subject considerable thought and investigation, concludes that mortuary reports are variable and cannot be accurately ascertained. Out of 7000 cases collected by him, the mortality varied from three to thirty-one per cent. "At the extremes of life it was very fatal, but benignant at intermediate periods, and complications of other diseases, chiefly phthisis and Bright's disease, greatly increasing it."*

No doubt the character and constitution of our patients, as well as their locality, have much to do in modifying our treatment. Patients admitted into our hospitals may have been poorly fed, clad, and cared for; are, perhaps, intemperate, or debilitated by previous habits of life, and the majority

* London Lancet.

even admitted after one or more lobes of the lung have become solidified. The physician may have indiscreetly based his therapeutics upon this class of patients alone.

It certainly requires but little discernment and judgment; for one to foresee and to decide that pneumonia thus occurring, indicates treatment diametrically opposite that called for in the same disease to be met with in the rural districts.

The cases I have herein noted occurred and were treated in what is sometimes called the "Genesee Country," whose inhabitants are mercifully doomed to earn their bread by the sweat of the brow, and by raising "Extra Genesee Wheat." Unlike those pent up in the confined air of an hospital, or those breathing the vitiated atmosphere of a crowded city, unaccustomed to sedentary life and late hours, they are of strong muscular fibre, full of blood, and inured to daily out-door labor. Consequently when they take on pneumonic inflammation, it must needs be of an active grade and of a sthenic character, calling for depletory measures.

Had my patients been subjected to the treatment advocated by Dr. Todd, to wit: ammonia and beef broth, they would have succumbed; or had they lived it would have been in spite of the disease or the expectant treatment. A like result would have ensued had I treated his patients by depletion, and withholding support. There is not so great a discrepancy in the treatment of a pneumonia as there is difference in the *character* and *type* of the disease.

*Dr. Burwell* then read his report as follows:

*Committee on Pneumonia.* Report of Dr. George N. Burwell.

I beg the indulgence of the society for not preparing any formal report upon the general subject of pneumonia. That prepared by the chairman of your committee (which I have not seen) will undoubtedly treat the subject in a manner much more fully and ably than I could hope to do; and should it give rise to discussion, it will then be easy to express my views upon any points upon which I may possibly hold opinions different from those advanced by the learned Professor.

As a substitute for a report of my own, I would now ask the attention of the society to the reading of two more cases which have occurred to me since our July meeting, together with a *resumé* of my experience with this disease for eighteen months past.

Case I. George McMichael, aged 5 years and 8 months, was taken sick at school, on Friday, July 20, 1855. He came home chilly; this was followed by fever, which had continued, without intermission, up to the time of my first visit on Sunday evening, July 22. He had then a pulse of 144, active and full; respiration 60; skin hot and dry; countenance anxious; had constant pain through his right shoulder, for which mustard poultices had been applied. He had had physic, which had operated freely before I saw him. On examination of his chest I found a slight dullness of percussion over the upper lobe of the right lung posteriorly. The respiratory murmur was dry and rude, with a rise in pitch. No positive bronchial respiration heard; no crepitus. Anteriorly (over the upper lobe right lung) the respiratory murmur was healthy, and percussion resonant. Elsewhere the lungs were sound.

I prescribed two grains each of calomel and Dover's powder, every two hours, until he should get easier, or his bowels move off again. To take a solution of nitre and soda for a drink.

George is a small, feeble boy. A month or two ago he weighed thirty-two pounds: deduct three pounds for his clothes, and it would leave him a naked weight of only twenty-nine pounds. He has, however, a good complexion, and has generally enjoyed good health.

July 23, A. M. The calomel has operated three or four times upon his bowels, with relief to his general symptoms. The local signs are as last night. Pulse fallen to 128, and the respiration to 40; has less heat of skin; still looks anxious and distressed.

To take an infusion of ipecac.

P. M. Pulse 120; respiration 50; has greater heat of skin than in the morning. The physical signs of pneumonia in the upper lobe, right lung, posteriorly, more distinct. There are traces of the bronchial respiration.

The ipecac. has not sickened him. Directed to double the dose, until vomiting is produced, and to give a Dover's powder at bedtime.

July 24, A. M. The boy rested tolerably well until two o'clock in the morning, when he became much worse. His fever is much higher this morning than at any time yesterday. Pulse 132; respiration 52, catching at the end of each inspiration, and consequently irregular and difficult to count. His cough distresses him greatly on account of the pain in his right shoulder and side, and he suppresses it as much as possible. His countenance is very anxious and distressed. Over the upper lobe, right lung, percussion is flat, posteriorly, and dull, anteriorly, and auscultation gives a very well-developed bronchial respiration, posteriorly, and rude respiration, anteriorly, showing that the inflammation commencing in or near the posterior surface of the lung, has extended itself anteriorly, so far as to involve, through and through, the entire lobe of the lung, and this with the positive development of pleurisy of the corresponding side.

Notwithstanding this amount of consolidation, and on the fifth day of the disease, I ventured to bleed. I bled until the pulse became softer and he became pale and faint. The amount of blood obtained was 4 oz. and 5½ drachms by weight. It was taken with the patient in a recumbent position. The vein was very small and the blood flowed mostly by drops; yet notwithstanding these unfavorable circumstances for the formation of a buffy coat, one was formed of nearly an eighth of an inch in thickness, and the crassamentum was so tenacious and elastic as to be pierced with a small round stick with difficulty, and not only could it be lifted out of the serum, but would bear the roughest shaking without tearing from the stick.

The pulse fell in frequency, instead of rising, on the occurrence of faintness. Directed a Dover's powder. To continue the ipecac. but not to nauseate him with it.

8, P. M. Pulse 132, has recovered its activity and firmness; respiration and other symptoms as in the morning; percussion quite flat anteriorly over the upper lobe, right lung, and the bronchial respiration is very distinct on auscultation; slight crepitation heard anteriorly at the end of inspiration.

The inflammation not having yielded in the least, and encouraged by the highly inflammatory character of the blood taken in the morning, I tied up his arm and took from the old orifice 2 oz. 2 drachms of blood. It flowed very slowly, drop by drop. This quantity softened his pulse and caused him to look pale, but did not produce faintness to the same extent as in the morning. Dr. J. C. Lay assisted me in the bleeding.

July 25, 8, A. M. The clot of the blood taken last night, was without any buff, but was firm and tenacious. The boy sweat after the bleeding, and rested tolerably well until one o'clock this morning, when the fever returned, and has since continued as high as ever. Pulse 132, again active and hard; respiration so constantly catching and irregular, as to make it impossible to count it with any accuracy. Coughs a good deal, and with very evident pain. His general appearance is as anxious and distressed as yesterday. The physical signs of the pneumonia remain very clear and well marked. Opened the vein anew and took 3 oz. and ½ drachm of blood (making exactly 10 oz. taken in twenty-two hours) which produced a decided effect on his pulse, and marked pallor of countenance and faintness.

Antimony was directed, in tenth of a grain dose, every hour, unless it nauseated him or operated on his bowels. Dr. C. C. Gay assisted me in the bleeding.

8, P. M. The clot of the blood taken this morning, was both buffed and cupped. The buff was not so thick as that obtained on the first bleeding, nor was the crassamentum quite so firm and resisting. His general appearance to night is decidedly that of being more easy and comfortable. There is less heat and fever; respiration still catching, and the cough is as painful as ever, although not as frequent. The antimony has not as yet sickened him or produced any sensible effect. I added one drop of laudanum to each dose, and directed it to be given every two hours.

July 26, A. M. Pulse 128; respiration 44, still catching, but less markedly so than yesterday. Did not have the usual exacerbation of fever last night; face cool; hands quite warm. There is an increase of the distinctness of the bronchial respiration, anteriorly; some crepitant rhonchus returning posteriorly, while the bronchial respiration there is much less distinct than on yesterday.

4. P. M. Pulse and respiration about as in the morning; some fever; cough continues quite painful.

9, P. M. About 7 o'clock this evening he got a severe pain, or colic, in his bowels, which caused him to make a great outcry. He simultaneously lost all traces of his fever. Has not had any movement of his bowels. I find his pulse fallen to 92; respiration easy; coughs but little. Directed six drops of laudanum for the pain in his bowels. Discontinue all medicines.

July 27. The boy has had a very comfortable night. Has not had any return of fever. Pulse 92, soft; respiration 32, with but little of the catch to it. The bronchial respiration, posteriorly, has nearly disappeared; anteriorly, it is quite distinct, but is not (and never has been) as distinct as it has been posteriorly.

July 28. The improvement continues. He had a hard coughing spell during the night, for which he took a Dover's powder (the last dose of medicine, of any kind, he had to take.) Pulse 96; respiration easy. The pleuritic symptoms have disappeared.

July 29. The boy's convalescence confirmed. The only physical signs of any inflammation having existed in his lungs are a dullness of percussion over the upper lobe, anteriorly, right lung, and a faint bronchial respiration heard on auscultation.

Doctors Hunt and Wyckoff also saw him in the course of his sickness.

August 20. I examined the boy's lung to-day. All traces of the dullness of percussion and bronchial respiration, noted in the last note, have disappeared, and he has recovered his weight (32℔) and has neither cough or other remnant of his sickness. His recovery has been complete.

In this case the patient was twice bled on the fifth day, and once on the sixth day (all three bleedings within twenty-two hours.) Duration of the disease, counting to the time the pulse fell below 100 and the fever permanently subsided, six days; or counting to the time of confirmed convalescence, eight days. One day at least was lost from neglect of prompt bleeding.

From the first bleeding to the permanent submission of the pulse and fever, two and a-half days; from the last bleeding to the same time, thirty-six hours.

Case II. *Lobar Pneumonia in a chill* 16 *months old, complicated with excessive tympanitis, and probable inflammation somewhere within the abdominal cavity.*

She was attacked Saturday night, July 21. I saw her the next day. She had fever and some cough, as also a scarlet rash over the face, neck and arms, which I thought might be scarlatina, more especially as vomiting was one of the first and most prominent symptoms on her being taken sick.

I directed merely a light febrifuge.

July 23. No more indications of scarlatina. The most prominent symptom was an excessive bloating of her bowels, which had come on since yesterday's visit. She had dyspnœa (which might well be caused by the tympanitis) and high febrile symptoms; pulse 160, active and wiry.

Directed three leeches to the chest, and laxative enemata.

July 24. The leeches bled only tolerably well, and without causing any reduction of the fever. No effect from the enemata. There is some vomiting; the bloating not reduced in the least. The abdomen has not the tenseness nor tenderness of peritonitis. The child's countenance and eye continues firm.

Two more leeches to be applied and the injections repeated.

July 25. Has less fever; is pale; countenance anxious and distressed; has constant hiccough and regurgitations of drinks; the tympanitis greater than at any time heretofore; no operations from the injections, the matters injected come away as given. Respiration and pulse both very rapid.

Gave no directions for further treatment, supposing the child to be very sure to die.

July 26. A diarrhœa came on spontaneously in the afternoon yesterday, which has reduced greatly the bloating, and with it the hiccough and regurgitation have ceased. The fever is higher; pulse 160, active; respiration

60; cough frequent, short and dry. Expression of countenance much better. Examined the lungs for the first time. Found an entire consolidation of the lower lobe, left lung, anteriorly, with by far the clearest, most distinct bronchial respiration I have heard this year. It was more like the cavernous respiration heard under circumstances very favorable for its production. There was also a fine and very abundant crepitus in both the lower and upper lobes. There were traces of the bronchial respiration in the upper lobe.

Directed three leeches and antimony, and a Dover's powder occasionally, if the cough becomes too harrassing.

July 27. A general amelioration of symptoms. Pulse fallen to 140; the respiration to 50.

July 28. An increase of fever to-day, with a rise of the pulse and respiration. A coarse crepitant rhonchus heard at the root of the right lung. Two more leeches directed. The bites of these were kept bleeding (and they bled unusually well) all day. At night I found the heat of skin less than on any day since I first saw her. The pulse was soft, but as usual in frequency. She was dreadfully pale, almost enough to frighten me. Her eye kept firm and good.

Sept. 4. I continued to visit my little patient daily for a week after the last note. There was no return or exacerbation of the inflammation to require any active treatment after July 28. The cough continued very troublesome for a fortnight after this date, with occasionally days of feverishness.

Dover's powders were given for the cough, and antimony whenever the fever appeared.

The bronchial respiration and the crepitant rhonchus, both disappeare. rapidly after the last leeching. The right lung fortunately kept free from the inflammation which threatened it. The respiratory murmur was slow in returning; but to-day I cannot detect any difference in the respiratory sounds in the two lungs. There is yet, however, a dullness of percussion over the left lung, posteriorly, as compared with the resonance of the right lung. The child has but little cough, is cheerful and playful, and has a great appetite. The diarrhœa has never stopped entirely, nor has the bloating of her bowels yet gone entirely away—nor will they, I think, before the setting in of cold weather. She continues pale.

I attended this patient daily, for a fortnight only, and made only one visit the succeeding week. I should be inclined to put down the duration of her sickness at about three weeks.

Before giving the *resumé* of my practice in pneumonia, for the past eighteen months, I would like to bring to the notice of the society, a few more statistics relative to the mortality of pneumonia as compared with other diseases.

1. In Massachusetts, the deaths from pneumonia, in 1853, were 884. The average number, for five years before and including 1853, were 885.

The number of deaths from pneumonia, in every 1000 deaths from all causes, were

| | |
|---|---|
| In 1849, | 42 |
| " 1850, | 52 |
| " 1851, | 50 |
| " 1852, | 45 |
| " 1853, | 45 |

Average for 12 years 8 months, 44; showing great uniformity in the mortality from the disease.

To show the true position of pneumonia, as a cause of death, in Massachusetts, I give the yearly average of deaths from the following diseases, for twelve years and eight months, viz:

| | | | | | | |
|---|---|---|---|---|---|---|
| From | Consumption, | 224 | in | every | 1000 | deaths. |
| " | Dysentery, | 75 | " | " | " | " |
| " | Fevers, | 70 | " | " | " | " |
| " | Pneumonia, | 44 | " | " | " | " |
| " | Scarlatina, | 42 | " | " | " | " |
| " | Croup, | 23 | " | " | " | " |
| " | Hydrocephalus, | 22 | " | " | " | " |
| " | Cholera Inf'm, | 20 | " | " | " | " |

2. *Kentucky.* In the mortuary tables of this state, for 1852, there is assigned to pneumonia 57 in every 1000 deaths, from ascertained causes, which it will be seen, is higher than for any year in Massachusetts for five years previous to 1854.

The same year (in Kentucky) the deaths from

| | | | | | | |
|---|---|---|---|---|---|---|
| Consumption | were | 92 | in | every | 1000 | deaths. |
| Dysentery, | " | 184 | " | " | " | " |
| Fevers, | " | 154 | " | " | " | " |
| Croup, | " | 44 | " | " | " | " |

3. *Rhode Island.* For the year from May 1st, 1852, to May 1st, 1853, the total number of deaths from known causes, were 1044.

| | | |
|---|---|---|
| Of these, - - - | 36 | died of Pneumonia. |
| " " - - - - - | 234 | " " Consumption. |
| " " - - - | 85 | " " Scarlatina. |
| " " - - - - - | 67 | " " Dysentery. |
| " " - - - | 39 | " " Hydrocephalus. |
| " " - - - - - | 35 | " " Cholera Infantum. |
| " " - - - | 30 | " " Croup. |
| " " - - - - - | 38 | " " Fevers. |

4. *Connecticut.* For three years (from July, 1848, to July, 1851) the deaths from pneumonia were in the ratio of 52 in every 1000 deaths.

| | | |
|---|---|---|
| From Consumption, | 213 | in every 1000 deaths. |
| " Dysentery, | 125 | " " " " |
| " Fevers, | 75 | " " " " |

5. *New Jersey.* For three years, from 1851 to 1853, there died

| | | |
|---|---|---|
| Of Pneumonia, in the ratio of | 28 | in every 1000 deaths. |
| " Consumption, " " " | 202 | " " " |
| " Dysentery, " " " | 103 | " " " |
| " Fevers, " " " | 25 | " " " |

The tables for New Jersey are said to be very imperfect, and, therefore, unreliable; those for Connecticut almost as much so; while those for Kentucky and Massachusetts, are quite reliable, especially those of the latter state. Those from Rhode Island have every appearance of care and reliability.

6. *The United States Army.* For the year ending June 30, 1854, there were 258 deaths in the army. Deduct those from epidemic cholera, yellow fever, and gunshot wounds, there would be left a number of 131 deaths.

There were treated during the year

| | | | |
|---|---|---|---|
| Of Pneumonia, - - | 62 | cases; of which | 6 died. |
| " Diarrhœa, - - | 2605 | " " " | 12 " |
| " Dysentery - - | 776 | " " " | 11 " |
| " Common Continued Fever, | 130 | " " " | none " |
| " Typhus Fever, - | 42 | " " " | 11 " |

From these figures we ascertain that the proportion of deaths from pneumonia, to the 131 deaths from ordinary causes, was in the ratio of 45 in 1000 deaths.

| | | | | | | |
|---|---|---|---|---|---|---|
| Of Diarrhœa | in the ratio of | | 90 | in 1000 deaths. | | |
| " Dysentery, | " | " | 84 | " | " | |
| " Fevers, | " | " | 84 | " | " | |

But of those attacked with pneumonia, there died in the ratio of 97 in every 1000 cases.

| | | | | | | |
|---|---|---|---|---|---|---|
| With Diarrhœa, - - | in the ratio of | | 4·5 | in every | 1000 | cases. |
| " Dysentery, - - - | " | " | 14 | " | " | " |
| " Fever (Typhus and Cont'd) | " | " | 64. | " | " | " |

7. In the private practice of *Dr. Brinsmade*, of Troy, N. Y.* He treated during the years 1853 and 1854

| | | | | | | |
|---|---|---|---|---|---|---|
| 58 | cases of | Pneumonia, - - - | of which | | 6 | died. |
| 752 | " | Diarrhœa, - - - - | " | " | 1 | " |
| 81 | " | Dysentery, - - - | " | " | 1 | " |
| 30 | " | Typhoid Fever, - - - | " | " | 3 | " |
| 45† | " | Croup, - - - - | " | " | 3 | " |
| 52 | " | Convulsions, - - - | " | " | 2 | " |
| 59 | " | Cholera Infantum, - - | " | " | 5 | " |
| 7 | " | Hydrocephalus, - - - | " | " | 5 | " |
| 7 | " | Congestion of the Lungs, - | " | " | 4 | " |
| 67 | " | Scarlatina, - - - - | " | " | 4 | " |
| 35 | " | Phthisis, - - - - | " | " | 19 | " |

Total deaths, in his practice, for the years 1853 and 1854—82.

| | |
|---|---|
| Ratio of deaths from pneumonia to total deaths, | 73 in 1000 |
| " " " " to cases of pneumonia, | 104 in " |

It will be seen that of the cases treated, only in phthisis, hydrocephalus, and congestion of the lungs, is the ratio of mortality greater than in pneumonia; and to the number of deaths phthisis only exceeds pneumonia.

In the following recapitulation I have made of cases of pneumonia, I wish to say that of none of them did I take notes except of the six, the histories

---

* See the Transactions of the New York State Medical Society, for 1855.

† Extremely impossible.—Ed. Jour.

I have had the honor of reading to the society, at this and previous meetings during this summer.

In the other cases I get the duration of cases by referring to my day book, where I find the time I attended in each case, and which will very nearly express the duration; for if the patients were generally sick two or three days before I was sent for, they were certainly fully convalescent two or three days before I fully discontinued my visits. On the point of treatment my memory is entirely reliable so far as stated in each case.

*Recapitulation.*

| | |
|---|---|
| Total cases of pneumonia, on my case book, for eighteen months, from March 1, 1854, to September 1, 1855, - - - | 52 cases. |
| Deduct cases seen but once or passing out of my hands, - | 9 " |
| Leaves, - - - - - - | 43 " |
| Deduct cases of lobular pneumonia (in children) - - | 19* " |
| Leaving of lobar pneumonia, - - - | 24 cases. |

For examination I shall divide these cases into the following four cases.

| | | |
|---|---|---|
| I. Typhoid Pneumonia, - - - - | 1 | case. |
| II. Sthenic " - - - - - | 13 | " |
| III. Complicated " - - - - | 6 | " |
| IV. Chronic " - - - - - | 4 | " |

In the class of sthenic pneumonia, are included three cases which partook more of the character of the congestive form of pneumonia, than of the sthenic—but are so placed to avoid too great a subdivision of cases.

I. The case of *Typhoid Pneumonia* was that of a hearty boy, 18 years of age, engaged in daily labor at the time of his attack. He had been sick three or four days when I first saw him. I prescribed the carbonate of ammonia, of which he took freely until his recovery. I attended him eight days. He was then fully convalescent, but remained feeble for some time afterward.

Duration of sickness, until convalescence, say twelve days.

---

* I propose for these a separate statement on some future occasion.

II. The sases of *Sthenic Pneumonia* I have tabulated as follows:

| Age. | Sex. | Duration of Sickness. | Treatment. |
|---|---|---|---|
| 4½ | M. | 9 days. | Bled once. See 2d case I reported in Aug. No. B. M. Jour. |
| 5⅔ | M. | 8 " | " 3 times in the 5 days. See 1st case rep'ted in this No. |
| 9 | F. | 9 " | " once. |
| 13 | M. | 6 " | Not bled. Dov. powder for cough; rest in bed; low diet. |
| 16 | M. | 8 " | Bled three times. 2d case in July No. Buff. Med. Jour. |
| 24 | F. | 6 " | " once. Physic, antimony. |
| 26 | M. | 9 " | " twice; cupped twice. Case complicated with pleurisy. |
| 27 | F. | 6 " | Not bled. Dov. powder; rest in bed. |
| 35 | M. | 9 " | Bled once, on the sixth day of the disease. |
| 37 | F. | 10 " | " twice, on the sixth and seventh days of the disease. |
| 37 | M. | 6 " | " once. 1st case in August No. of Buff. Med. Jour. |
| 39 | M. | 12 " | " three times. 1st case in July No. of Buff. Med. Jour. |
| 43 | M. | 10 " | " once, the fourth day of the disease. |

III. Cases of *Complicated Pneumonia.*

| Age. | Sex. | Complication. | Duration. | Treatment. |
|---|---|---|---|---|
| 16 mos. | F. | Tymp'itis and probable inflammation within abdomen. | About 3 wks. | See second case reported this evening to the society. |
| 25 years. | M. | Delirium tremens. | 32 days. | Bled once. Left house the day after the bleeding, and wandered miles over the city. |
| 27 years. | F. | Pleurisy. Miscarriage. | 9 days. | Bled once, but could not get over 4 or 5 oz., which had only a thin buffy coat, and a friable clot.—Was cupped. Miscarried, with great suffering, the 7th day of the disease, and then sunk into a cholera-like collapse, and died in 48 hours afterward. Had quinine and stimulants after the bleeding. |
| 32 years. | F. | Rheumatism. Pericarditis. | 22 days. | Bled twice. Blood very buffy and with a firm clot. Recovery perfect. |
| 45 years. | M. | A constant and severe hiccough during the last week. | 22 days. My attendance was 15. | Was sick a week before I saw him, and traveled by railroad over 1000 miles during that week. Had no treatment before he stopped in Buffalo. Bled him with the best effects, on the 9th and 12th days of the disease. Vesication. Had it not been for the hiccough, my attendance would have been nearly a week short of what it was. |
| 54 years. | F. | A chron. cough with a constant loss of flesh and general deterioration of health for over 6 months previous to illness. | 33 days. | Bled once on the 3d or 4th day of the disease. Anodynes; vesication; expectorants, and finally tonics and cod liver oil. |

IV. Cases of *Chronic Pneumonia.*

| AGE. | SEX. | GENERAL HISTORY. |
| --- | --- | --- |
| 2 years. | F. | Was taken sick six weeks before I saw her. The only treatment she had had was some medicines, occasionally, of a "German physician." On my first visit, bronchial respiration was still present and distinct in the lower lobe of the right lung. She was having exacerbations of fever every afternoon. Treated with quinine. Improvement rapid for awhile. Is still liable to catch cold easily. |
| 4 years. | M. | Sick ten days before I saw him. Had not had any treatment except that directed by his mother. Bronchial respiration was still present in the upper lobe of the right lung. I gave physic and antimony, as there was still some excitement of the circulation. After three days' attendance he was well enough to dismiss. |
| 6 years. | F. | Was taken sick five months before I saw her, with fever and cough. Was confined three weeks to her bed and two months to the house. Has since been very subject to colds, that is, to turns of high fever, with a cough. These attacks confine her three or four days at a time, and have recurred every two or three weeks, up to the time of my visit, five months after the original attack. On examination of her lungs, I found a marked dullness over the upper lobe of the right lung, anteriorly, and a feeble blowing sound in place of the normal respiratory murmur. There were none of the rhonchi to be heard. The child is otherwise lively and well; does not get fleshy. At the time of her first attack she was attended by a physician, but was not bled or leeched. |
| 40 yrs. | F. | Catharine McGinn, aged 40 years; never before sick in her life, except at her confinements. Was taken with a chill on the 6th of July last; was very chilly all night; says she felt no heat after the chill. The next day (July 7th) a cough commenced, which has continued to harrass her ever since. She spit twice of blood on July 7th, the only time she has ever raised any. She coughed very badly for some days; the |

expectoration was tough and sticky, more so than at present (July 25th, 1855.) She has had continued thirst from the initial chill, with loss of appetite and great loss of flesh.

She entered the hospital, July 25th. Her pulse was 88; respiration moderately hurried; heat of skin considerable; cough troublesome and paroxysmal; expectoration muco-purulent; complains of pain in the left side.

On a physical examination of the left lung, I found percussion flat over nearly the whole of the lower lobe, posteriorly, and in the lower two-thirds of the axillæ. No respiratory murmur to be heard in the lower lobe, nor bronchial respiration or broncophony. There is a mucous rhonchus, mixed with a coarse crackling or crepitation. In the upper lobe the respiratory murmur is feeble and mingled with both the mucous and sibilant rhonchi. Percussion normal.

In the right lung, percussion is dull at the base, posteriorly —it is clear in the axillary region, normal over the upper lobe. The respiratory murmur is distinctly to be heard throughout lung except in the lower half (posteriorly) of the lower lobe, where it is quite feeble. She had not had any medical treatment. She remained in the hospital some three weeks, and then left, without experiencing any very material improvement.

In this connection I would take the liberty of reading a short note of a case of asthma, having its origin in a pneumonia which was allowed to run its course without bleeding or other effective treatment, and is, therefore, pertinent to our subject.

Mr. Asa Bailey, aged 36 years, residing in the town of Hamburgh, and a blacksmith by trade, came to consult me in May last, for difficult breathing. His account of himself was, that he used to have attacks of "asthma," whenever he got cold, from early childhood until he was twelve years old. From that time he never was sick until the present illness, was always strong and hearty, perfectly free in his wind, and without a symptom of lung disease.

In October, 1852, he got sick with a cough and fever which confined him some days to his bed. Not getting along well he came to Buffalo, sick as he was, to see a Thompsonian physician, and he recollects well while here in Buffalo, of coughing badly and spitting blood. He remained sick and confined to his house all that fall and winter. In the spring of 1853 he commenced doing some work, but feebly and at intervals. He has never been free from cough, and shortness of breath, since he got sick. His expectoration is scanty and greenish. Is the thinnest in flesh he has ever been since he grew to manhood. None of his immediate relatives have ever died of consumption. His chest is generally clear on percussion; the respiratory murmur is unusually feeble, imperfect and wheezing.

In the resolution referring the subject of pneumonia to the committee, was a clause instructing said committee to report upon "the diagnostic value of the buffy coat;" in other words, asking for a statement of the differences (if any) between the blood drawn in pneumonia and that drawn in other diseases. To make such a statement requires many observations of the appearances of the blood in a great variety of diseases. I know of none made directly upon this point; but it is worthy of attention, and I would here make a smail contribution to this object. It is an account of the appearance of the blood drawn in four cases of patients not suffering from pneumonia. I have already given in the histories of cases, an account of its character in five cases of this disease. The observations extend only to the variations of the appearances of the buffy coat and of the firmness and tenacity of the crassamentum. The actual and comparative weight and quantity of the clot and serum in the different cases, would be interesting subjects of observation, but which I have neglected to do.

1. In July last, I bled a hearty, healthy woman, 22 years of age, in labor with her first child, for rigidity of the os uteri. About 22 oz. were taken. The clot had a li_ht covering resembling the buffy coat. It tore easily and was composed of two very thin, delicate membraneous layers, with a cellular structure between them. The upper layer could be lifted off or slid one side without disturbing the lower layer. The cellular structure between the layers was irregular, with a transparent serum in its meshes. The crassamentum had only a moderate consistence, and tore easily on pushing a stick through it.

2. In July, in consultation with Dr. Wilcox, I bled a man, about 27 years of age, with chronic peritonitis complicating other diseases of the bowels. The quantity taken was about eight ounces. It had a substitute for the buffy coat in a thin bluish semi-transparent covering of illy-organized lymph, wholly without strength or tenacity. The clot was very dark, soft, and tearing readily on the least attempt at lifting it up.

3. Last month I bled a woman, 28 years of age, for symptoms of peritonitis after miscarriage. I took about 16 oz. The center of the surface of the clot had a distinct buffy appearance; the rest of the surface had only a very slight trace of it. The crassamentum had a certain degree of tenacity, but would not lift out of the serum without tearing.

4. About the same time I bled a man 34 years of age, for peritonitis complicating an obstruction of the bowels. I took 20 oz. It had a well-formed, firm buffy coat, an eighth of an inch thick, such as is so often seen in sthenic pneumonia. But unlike the blood in pneumonia the clot was exceedingly soft, not being more tenacious than a mass of jelly.

*Dr. Flint* followed Dr. Burwell in the annexed

*Report of the Chairman of the Committee appointed by the Buffalo Medical Association, June 3d, 1845, in conformity with the following motion:*

"That the subject of pneumonia, its pathology, prognosis and treatment, together with the diagnostic value of the buffy coat, and also the papers read upon this subject at this and the preceding meeting, be referred to a committee of three to report at the next meeting."

The resolution specifies three distinct aspects under which pneumonia is to be considered, viz., its pathology, prognosis and treatment. It also refers to the committee the papers relative to the treatment of the disease, read at the meetings of the association in June and July last. And, in addition, another subject is included, viz., the "diagnostic value of the buffy coat."

On consulting as to the mode of discharging the duty entrusted by the association, the committee agreed that each member should draw up a report separately, to be presented without reference to those of his colleagues. This plan, it is proper to state, was adopted, not because, so far as compared, the views entertained by the different members of the committee were discordant, but on account of the inconvenience, and, indeed, impracticability, at this season, of holding meetings for a full discussion of the various questions which would be likely to arise in connection with the subjects embraced in the resolution; and a reluctance on the part of the chairman of the committee to ask his colleagues to unite in a report without, on their part, sufficient time and opportunity to give to it a thorough examination, which, owing to the brief period allotted for its preparation, would be impossible. For the views, therefore, contained in the following report, the chairman is alone responsible; and it is presented to the association without having been submitted previously to the committee.

In treating of pneumonia under the three aspects specified in the resolution, I shall assign to each a separate division, taking them up in the order in which they have been already enumerated, viz: 1st. The Pathology; 2d. The Prognosis, and 3d. The Treatment.

1. *Pathology.* The pathology of pneumonia, claims but a brief space in this report. The doctrines which, in the existing state of medical science, pertain to this aspect of the disease, are, in general, held by the profession with unanimity, and are sufficiently familiar to the members of this association. I shall limit my few remarks mainly to certain pathological facts and distinctions important to be premised before directing attention to the prognosis and treatment of the disease. Pneumonia is presented, clinically, as a primitive and a secondary affection. This is a very important distinction, not merely in a scientific, but in a practical point of view. As a secondary affection, it is apt to occur during the progress of typhoid and typhus fevers, and the exanthematæ, especially rubeola; in pyæmia, rheumatic fever, croup, and glanders. Another important distinction relates to its existence alone, or in association with other local affections. Thus it may coëxist with pericarditis, with organic disease of the heart, and with Bright's disease. The inflammation may be limited to the pulmonary parenchyma, or, in conjunction, other anatomical structures entering into the composition of the lungs may be implicated. In the vast majority of the cases occurring in the adult, as is well known, pleuritis exists as a complication. In children bronchitis is generally associated, constituting what is distinguished as broncho-pneumonia; on the other hand, the combination of pneumonia and general bronchitis is only an occasional coincidence in the adult. The associated bronchitis is sometimes of the capillary variety, and the disease then presents features widely different from those incident to its concurrence with bronchial inflammation confined to the larger tubes. The relations of age to the distinctions just mentioned, and some others, constitute not only an interesting, but a highly important part of the pathological history of pneumonia. In the aged, as in young subjects, the disease, in a very large proportion of cases, is developed in combination with bronchitis, a combination adding largely to the danger. It receives very important modifications from the coöperation of causes of disease peculiar to certain localities. In districts where intermitting and remitting fevers are rife, the phenomena due to the special cause of these affections are so generally intermingled, that a current notion in many parts of our country ascribes the pneumonia to a miasmatic origin. It may prevail as an epidemic, and in this form is subject to the variations to which all epidemic affections are liable. And irrespective of well-marked epidemic influences, it presents, at different times and places, differences affecting materially the prognosis and treatment. Like most acute diseases the gravity, and, measurably, the characters of this have relation to the previous health, the constitution and the habits of the patient. Occurring in the intemperate it is joined in its march not infrequently by delirium tremens, an important union as respects the probable issue and the management. It is unnecessary to extend this enumeration to show that, in a great measure the semeiological history of cases of pneumonia, but more especially the gravity of the disease, and therapeutical indications are dependent on a variety of contingencies, and that in certain of its forms and combinations, the pneumonia, so far as concerns its relations to the prognosis, the treatment, and the symptoms also, is itself the contingent circumstance. It is superfluous to add that in this respect the disease is not peculiar, the same being true, to a greater or less extent, of most acute local inflammations.

A few additional pathological points which relate, not to incidental circumstances, but to the disease itself, may with propriety be premised.

Pneumonia, occurring as a primitive affection, is generally abrupt in its access. The inflammation in the adult, in the great majority of cases, extends over an entire lobe at least, constituting lobar pneumonia. When two or more lobes are attacked, they are invaded, not simultaneously, but successively. The lower lobe is much more prone to become inflamed than the upper; and the affection is oftener limited to the right than the left side. Inflammation of the upper lobe (which is more likely to occur in the aged) is a graver form of the disease than when it is limited to the lower lobe. Double pneumonia, i. e. the inflammation seated in both sides of the chest, is exceedingly rare in the adult.

Inflammatory exudation within the air cells inducing solidification, more or less complete, of the portion of the pulmonary organs over which the inflammation extends, occurs in so large a proportion of cases that, practically, it may be considered to be an uniform result. This result generally follows quickly on the stage of invasion, and constitutes the second stage of the disease. The physical evidences of solidification may be present in a few hours after the attack; and the instances are small in number in which they are not apparent within the first five days. The disappearance of inflammation, therefore, by delitescence, i. e. without the deposit of inflammatory products, is not to be expected.

In its progress and tendencies, pneumonia differs from acute inflammation affecting some other structures, in several striking particulars. It generally runs a rapid career; the local anatomical changes marked as they are, incapacitating the affected portion of lung temporarily for the performance of its function, follow in quick succession. The liability to end in chronic inflammation is exceedingly slight. The formation of abscesses is also extremely rare. When pus is formed, it is generally infiltrated, not encysted; termination in gangrene occurs in but a very small proportion of cases. Relapses, after convalescence is established, are infrequent. As regards the points just mentioned, the laws of the disease are eminently conservative, contrasted with the destructive effects so often incident to the processes of inflammation.

Lobar pneumonia, the form the disease assumes (as already stated) in the adult, is also presented not infrequently in children. It occurs in early life oftener than has of late years been generally supposed. But another form is peculiar to infantile life to which allusion has been already made, viz., broncho-pneumonia, or lobular pneumonitis. This form of the disease offers so many points of contrast to lobar pneumonia that it hardly belongs in the same nosological category. Broncho-pneumonia, indeed, may with greater propriety be considered a variety of bronchitis, than of pneumonia; and it has been proposed to apply to it a title expressive of this fact, but at the same time, involving a solecism, viz., vesicular bronchitis.

In these few remarks under the head of the pathology of pneumonia, I am well aware nothing has been mentioned which possesses novelty for the members of the association. My object has been simply to adduce, in as brief terms as possible, certain pathological truths, to some of which I may have occasion to refer in the remarks which are to follow, and of which it is more convenient beforehand to refresh the memory.

2. *Prognosis.* My remarks under this head will have reference mainly to the following inquiry: What amount of danger to life does pneumonia involve? Judging from the tenor of the papers referred to the committee, and the discussions growing out of them at the meetings of the association, this question covers sufficient ground to meet the objects for which the prognosis was specified in the resolution. A little reflection will suffice to show that the inquiry is not to be answered simply by collecting mortuary statistics. Dead *with* pneumonia is one thing, and dead *from* pneumonia is quite another thing. The question is not, 'of the number of persons who die within a given time, how many were affected with pneumonia?' but what is the intrinsic tendency of the affection, disconnected from complications, associated morbid conditions, and incidental circumstances which exert influences more or less powerful in determining a fatal result? We cannot expect to settle this point with rigorous precision; all that we can do is to arrive at conclusions approximating to exactness.

It has been seen that pneumonia occurs secondarily in connection with diverse diseases. Grisolle lays down the principle that it is liable to become developed whenever high febrile movement exists, conjoined with feebleness of the vital forces. On the other hand, clinical observations have abundantly established the fact, first pointed out by Piorry, that a low grade of inflammation, in a certain proportion of cases of various chronic or protracted diseases, is developed toward the close of life as the result of passive and even hypostatic congestion, often proving the immediate cause of death. In nearly all cases of the latter description, and in a large proportion of the instances in which the disease is secondarily developed in the course of typhus, typhoid and remitting fevers, and the exanthematæ, the issue is fatal. But, plainly, the rate of mortality under these circumstances is not to be considered in the light of data for estimating the fatal tendency which belongs to primitive, uncomplicated pneumonia.

Age is to be ranked among the incidental circumstances influencing the result in cases of pneumonia. In very young infants the disease proves fatal in the great majority of instances. At the *hôpital des enfants-Trouvés*, at Paris, it is found that children attacked shortly after birth are uniformly destroyed. In the aged the fatality is very large. At the *hospice salpêtrière*, in Paris, an institution for the reception of aged females, containing several thousand inmates, in 1841, one-half of those who were affected with the disease perished; and during some years, it is stated by Valleix, the rate of mortality has been still greater. It is evident that the proportion of fatal cases at the two extremes of life is not a fair criterion for determining the prognosis during the intermediate period. Pneumonia occurring in the intemperate is an affection of unusual gravity; and the same remark is applicable to the occurrence of the disease in persons of feeble constitution, as well as all cases in which from deprivation, over exertion, or other causes, the ability of the system to bear up under any acute affection is impaired. These considerations will prepare us to appreciate the application of mortuary statistics in endeavoring to arrive at correct notions relative to the amount of danger to life which pneumonia intrinsically involves.

In the paper read to the association by a member of the committee, at the meeting in July, statistics of the ratio of deaths from pneumonia in Philadelphia, New York, and our own city were given. Through the kind assistance of a member of the association, Dr. John Root, I am able to embrace in this report the proportion of deaths attributed to inflammation of the lungs, to the whole number of deaths from all diseases, in the city of New York, from 1804 to 1853, inclusive, a period of fifty years. These statistics have been calculated by Dr. Root from data contained in the annual report of the city inspector for 1853.

*Proportion of deaths from Pneumonia to deaths from all diseases in the city of New York, from* 1804 *to* 1853, *inclusive.*

| | | | |
|---|---|---|---|
| 1804, | 20 | in | 1000 |
| 1805, | 47 | " | " |
| 1806, | 59 | " | " |
| 1807, | 61 | " | " |
| 1808, | 51 | " | " |
| 1809, | 52 | " | " |
| 1810, | 65 | " | " |
| 1811, | 42 | " | " |
| 1812, | 91 | " | " |
| 1813, | 90 | " | " |
| 1814, | 35 | " | " |
| 1815, | 84 | " | " |
| 1816, | 81 | " | " |
| 1817, | 60 | " | " |
| 1818, | 54 | " | " |
| 1819, | 47 | " | " |
| 1820, | 43 | " | " |
| 1821, | 44 | " | " |
| 1822, | 63 | " | " |
| 1823, | 54 | " | " |
| 1824, | 53 | " | " |
| 1825, | 69 | " | " |
| 1826, | 56 | " | " |
| 1827, | 53 | " | " |
| 1828, | 48 | " | " |
| 1829, | 65 | " | " |
| 1830, | 42 | " | " |
| 1831, | 64 | " | " |
| 1832, (cholera year) | 42 | " | " |
| 1833, | 76 | " | " |
| 1834, (cholera year) | 62 | " | " |

| | | | |
|---|---|---|---|
| 1835, | 71 | in | 1000 |
| 1836, | 73 | " | " |
| 1837, | 76 | " | " |
| 1838, | 79 | " | " |
| 1839, | 69 | " | " |
| 1840, | 72 | " | " |
| 1841, | 78 | " | " |
| 1842, | 68 | " | " |
| 1843, | 75 | " | " |
| 1844, | 69 | " | " |
| 1845, | 75 | " | " |
| 1846, | 55 | " | " |
| 1847, | 54 | " | " |
| 1848, | 50 | " | " |
| 1849, | 42 | " | " |
| 1850, | 61 | " | " |
| 1851, | 64 | " | " |
| 1852, | 54 | " | " |
| 1853, | 51 | " | "* |

The above table gives 61 deaths in 1000, as the average for fifty years. The greatest ratio in any one year was in 1812, being 91 in 1000. The next highest was in 1813, when it was 90 in 1000. These years are noted in history for the extensive prevalence of an epidemic considered to be typhoid pneumonia. The smallest ratio was in 1804, when it was only 20 in 1000. The whole number of deaths from all diseases during the fifty years, was 333,819. Whole number of deaths attributed to pneumonia, 20,363.

In Boston during a period of five years, the proportion of deaths attributed to pneumonia, is 1 in 44 deaths from all diseases, i. e. a fraction less than 93 in 1000.

In Charleston, S. C., 1 in 69, i. e. a fraction under 14 in 1000.

In London, 1 in 11, i. e. 90 in 1000.

These statistics show a very considerable variation in the relative proportion of deaths from pneumonia in different localities, and, also, in the same locality in different years: but the ratio of mortality ascribed to the disease is uniformly large. According to Dr. Walshe, taking the returns made to

---

* Still-born deaths are excluded.

the registrar general of Great Britain, in 1839, as a fair average, this inflammation would appear to hold the third place among the fatal diseases.

Calculating from the data afforded by the mortuary reports of the city of New York for fifty years to 1853, inclusive, it ranks second in the list. For the calculations on which this statement is made, I am indebted to Dr. Root.*

Now with reference to the amount of danger to life which pneumonia involves, what value is to be attached to the foregoing statistics? This is a point which has given rise to discussion in the association, and its bearing on the subject of the next division of the report, viz., the treatment, is manifestly important. It is to be borne in mind that our present object is to endeavor to determine the intrinsic tendency of pneumonia to a fatal result, disconnected from complications, associated morbid conditions and incidental circumstances. Mortuary statistics are, of course, available for this object only so far as they relate to cases of pure, idiopathic, uncomplicated pneumonia; occurring between the extremes of life, and under circumstances not unfavorable to the successful resistance of disease. What proportion of the cases among the number of reported deaths with this disease embrace the conditions just named, it is impossible to determine with exactness; but there are reasons for believing that in the vast majority of instances these conditions are wanting. A large share of the deaths purporting to be from pneumonia, are among children and the aged. This fact can be shown by statistics. Dr. Swett, in his treatise on diseases of the chest, gives the ages in the fatal cases of pneumonia occurring in New York during the years 1848, 1849, and 1850. The whole number of reported deaths from pneumonia during these three years, was 2449. Of this number, 1420 were of children under five years of age; and 206 were of persons over fifty, leaving 823 the number of fatal cases occurring between the ages of five and fifty. By this process the number of cases is reduced two-thirds. In forming an idea of how large a share of the number of cases which remain, were cases of pure, idiopathic, uncomplicated pneumonia, occurring under circumstances not unfavorable to the successful resistance to disease, we are to make allowance for carelessness in the collection of statistics; for errors of diagnosis, and a host of incalculable unfavorable circumstances, pertaining to individual cases. There are no means of determining directly how many cases would be eliminated

* The deaths from the eight most fatal diseases, are as follows: Consumption, 56,113. Pneumonia, 20,363. Convulsions, 18,256. Cholera Infantum, 14,685. Dropsy of Brain, 14,387. Dysentery, 11,417. Typhus and Typhoid Fevers, 11,282. Croup, 8,535.

were we able to subject them to this analytical process; but that the balance would be small, we may conclude by an indirect method of investigation.

If we take the results in private or public practice reported by different persons in whose good faith and ability as diagnosticians confidence may be placed, the rate of mortality offers a striking contrast with that of the mortuary statistics which have been cited. These results show that the proportion of instances in which idiopathic, uncomplicated pneumonia proves fatal under different methods of management, and even without any active therapeutical interference, is small. And in these results allowance is not made for unfavorable circumstances, such as previous ill-health; feeble constitution; impaired ability to resist disease, from deprivation, want of proper care, etc. Giving due allowance to these circumstances, I think we shall be warranted in concluding that pneumonia intrinsically tends to recovery; in the vast majority of cases of death *with* pneumonia, the fatal issue being due to conjunctive, contingent causes. This conclusion accords with the conservative laws of the disease referred to in the previous division of this report.

I will give, first, the results as respects fatality, in my own experience. Among my hospital notes I find recorded the histories of thirty-eight cases entitled pneumonia. Of these thirty-eight cases six proved fatal. On examination of the histories of the fatal cases, in one, death occurred on the second day after admission; the physical examination was partial, no autopsy was had, and it is not positive that the pneumonia was idiopathic and uncomplicated. In another case death occurred between seven and eight hours after admission, and there was no autopsy. The same objection will, therefore, apply to this case. In a third case, a post-mortem examination revealed the coëxistence of pericarditis, which was not discovered prior to death. Excluding these three cases, three remain. In all these cases the chest was examined after death, and in all the pneumonia was double. In two, the lower lobe of the left lung was in the third or purulent stage; and the lower and middle lobe of the right lung in the second stage. In the other case the inflammation was confined to the lower lobes; that of the right lung being in the third, and that of the left in the second stage. From this collection are excluded cases occurring in childhood or old age, and all cases save those of primitive, uncomplicated pneumonia. The proportion of deaths is three in thirty-five cases. Of cases occurring in private practice I have preserved notes of but few; but I believe I am correct in stating that exclusive of cases occurring in children and the aged, I have not met with a single instance of a fatal result from primitive, uncomplicated pneumonia during the last six years.

The prognosis in individual cases of pneumonia, aside from all extrinsic or contingent circumstances, it is evident must have reference, among other points, to the extent to which the lung is involved. In the three hospital cases in which the result was fatal, and post-mortem examinations made, the existence of double pneumonia was verified. Double pneumonia, it is well known, is a dangerous form of the disease. Excluding these cases, and I have not an instance of death from primitive, uncomplicated pneumonia. Yet, in several of my hospital cases the inflammation was either confined to the upper lobe, a graver form of the disease than when limited to the lower lobe, or it extended over an entire lung, a form of the disease still more grave. In view of these facts, judging from my own experience, I should say that primitive, uncomplicated pneumonia, in the adult, confined to a lower lobe, as is the case in the vast majority of instances, invariably ends in recovery. In this conclusion it is of course assumed that the disease is sporadic, that it occurs in a non-malarious district, and under circumstances pertaining to constitution, etc., favorable for passing through with safety any acute disease.

The data furnished by my own experience, however, are quite insufficient to establish the foregoing conclusions. The limited number of hospital cases which I have collected, and still more the cases occurring in private practice, it may be said, by a series of coincidences, were of an unusually favorable character. Let us, then, see how the results compare with those, on a larger scale, gathered from different sources.

In an able article communicated by Dr. Ames, of Montgomery, Alabama, for the New Orleans Medical and Surgical Journal, January, 1854, the author has tabulated the cases occurring in his practice, from 1849 to 1853, inclusive. The whole number of cases is sixty-eight, but thirteen were cases occurring under five years of age. Exclusive of these, the number is fifty-five. Of these fifty-five cases one only was fatal. This was a case of double pneumonia, the lower and middle lobes of the right lung being affected. The collection of cases embraces seven additional instances in which the pneumonia was double. In a number of instances the upper lobe was affected; and diverse unfavorable circumstances are mentioned, under the head of remarks, as incidental to several of the cases. These results are from a portion of our country where the disease prevails in an epidemic form, associated, frequently, with phenomena distinguished as miasmatic.

I may remark here, that Dr. Ames adduces his cases in illustration of certain views respecting the treatment of the disease. How far peculiarities of treatment may have had influence in determing statistical results, I shall not stop to inquire. In connection with the subject of treatment I shall refer again to the statistics presented under this division of the report. Inasmuch, however, as the supposed influence of the treatment may be considered to be an important point of inquiry in determining the fatal tendency which belongs intrinsically to the disease, it should be stated, and it has been already intimated, that the statistics which I shall cite will embrace cases treated differently, and also without any active remedies. I may add that in the treatment of the cases reported by Dr. Ames, neither bleeding, antimony nor mercury entered.

Adducing, next, statistics, not of the experience of one practitioner, but containing the aggregated results in a large number of cases treated in different countries, the official returns of the British army show among the troops stationed at Gibraltar, Malta, Ionian Islands, Bermudas, Canada, Cape Mauritus and St. Helena, 12,271 cases of pneumonia.* Of this large number of case, 413 were fatal; a proportion of 1 in every 29, or about 3 per cent.

Now, of a given number of cases, is it not fair to presume that in the proportion of three per cent. intemperance or other unfavorable circumstances, or certain complications such as pericarditis, Bright's disease, tuberculosis, would be likely to coëxist and determine a fatal result? If, in addition to this consideration, we bear in mind that double pneumonia occurs in a larger proportion than 3 per cent., do not the results just cited go to show that simple, uncomplicated idiopathic pneumonia in the adult when limited, as it generally is, to a single lobe, is intrinsically devoid of any tendency to a fatal result?

The official navy returns, in Great Britain, show 3,099 cases of pneumonia in different parts of the world. Of these, 136 were fatal; a proportion of 1 in 23, or 4 per cent. These results correspond, in a striking manner, with those obtained from the army returns; a fact going to show that they indicate a law of the disease as regards its fatality.

So far as treatment is concerned, army and navy returns being received from a great number of practitioners, probably embrace cases treated in various, and, perhaps, opposite modes. But I shall now adduce the results in a large number of cases in which no active treatment was adopted. At the great hospital at Vienna, 750 cases of pneumonia were received from 1847 to 1850. The treatment in all was limited to gum water, and *tisanes* containing opium. Of these cases sixty-nine were fatal; a proportion a little

---

* For the statistics derived from the British army and naval returns I am indebted to Prof. Simpson's work on homœopathy. The period within which the cases occurred is not given.

over 9 per cent.* The percentage of deaths is here considerably larger than in the army and navy practice of Great Britain; but the Vienna collection, it is distinctly stated, includes cases in which the pneumonia was secondary. It is also stated that none of the fatal cases were exempt from complications. Assuming these statements to be correct, the results are fully in accordance with those previously cited.

I might multiply citations of statistical results, but I am fearful of wearying the patience of the association. I will content myself with adding the account which the great Laennec gives of the fatality attending the disease in his experience. Laennec advocated a single moderate bleeding in the treatment of pneumonia, and afterward reliance on antimony after the Italian method introduced by Rasori. Laennec states† that in 1824, at the clinic of the faculty of medicine, he treated twenty-eight cases, in all of which recovery took place with the exception of one cachectic old man of seventy. In 1825 he treated thirty-four cases, of which five died. But of the five fatal cases, in three the ages were 50, 69, and 70, and two were brought into the hospital moribund. Of the two remaining instances of death, one died from a complication of heart disease, and the other of chronic pleurisy supervening on resolution of the pneumonia.

3. *Treatment.* Inasmuch as the papers presented to the association, and the discussions therewith connected, have had relation solely to bloodletting in pneumonia, I shall limit my remarks under the head of treatment, to the employment of that therapeutical measure.

In determining the importance of active interference in the management of any disease, a point first to be considered is the intrinsic tendency of the disease to recovery or otherwise. It may be enunciated as a maxim in therapeutics, that the necessity for powerful remedies in the treatment of any disease is inversely in proportion to its tendency to recovery. This maxim is correct as a general rule; but the converse does not hold good. The urgency or propriety of active interference is by no means always proportionate to the fatal tendency of a disease. There is another point of view in which the intrinsic tendency of a disease to a favorable issue is an important element in therapeutical investigations, viz., the proportion of recoveries is, of course, not a test of the success of particular methods of treatment. This point is often overlooked by medical practitioners. It happens not infre-

* Virginia Journal of Med. and Surg. 1853.

† Treatise on Diseases of the Chest.

quently (and the literature of the disease under consideration furnishes an illustration of the remark) that methods of treating a disease directly opposite in their character, are advocated on the strength of uniform or an equal amount of success as indicated by the proportion of recoveries. Under these circumstances it is fair to infer generally that the recoveries were due to the intrinsic tendencies of the disease. In the instance of a disease which tends almost uniformly to end in recovery, while a large ratio of fatal cases affords strong evidence of the destructive influence of potent measures which may have been employed in the management, conversely the result furnishes no evidence of the efficiency of treatment, because, irrespective of any interference, the disease would not be expected to end fatally.

Applying these general principles to the present subject, viz., the treatment of pneumonia by bloodletting, it has been seen that uncomplicated and idiopathic pneumonia, occurring under circumstances not unfavorable to recovery from any acute affection, rarely proves fatal under different modes of treatment, and even without any active interference. The fact, then, that patients generally recover when bleeding is practiced, does not prove that this measure contributes to that result, although, on the other hand, the non-recovery of a number considerably greater than the average ratio of deaths might constitute strong presumptive evidence of the pernicious effects of this mode of treatment.

Has the employment of bloodletting been found to increase the mortality in cases of pneumonia? I am not prepared to give an answer to this question based on statistical researches; but in view of the great extent to which bleeding has been practiced in this disease from the earliest period in the history of medicine, it is, perhaps, reasonable to conclude that its influence on the fatality has not been conspicuously marked. In fact, it is well known that pneumonia is an affection in which bloodletting is well borne, so far as its immediate effects are concerned. Bouillaud, the clinical professor at *La Charité*, at the present time the most ardent of the advocates for bloodletting, has formularised the use of this measure, laying down explicit rules designating the number of bleedings, and the extent to which they are to be carried in all cases indiscriminately. The mean quantity of blood detracted in individual cases, in following out his formula, is about four pounds five ounces. The blood is removed in small quantities at a time, by general bloodletting twice daily, or alternating with local bleedings by cups or leeches, the repetitions being continued till the acute local symptoms and the febrile movement cease. This is the *coup-sur-coup* system of bloodletting. Bouillaud claims by this method to effect a larger number of cures, and has published statistical results in evidence of the superiority of his practice. These results show a mortality of one-eighth,* which certainly cannot be cited as evidence of extraordinary success. Not being able to refer to Bouillaud's publication, I am not aware of the circumstances connected with the fatal cases which it may be important to take into account in endeavoring to estimate the influence of the treatment. In general, the objections brought against profuse bloodletting in this disease by those who advocate its moderate employment, or the substitution of other measures, relate not so much to the immediate danger from the remedy, as to its ulterior effects in retarding convalescence, and impairing the health after recovery from pneumonia. Dr. Walshe remarks, in connection with this subject, that in cases of this disease "in which blood has been too lavishly sacrificed, a form of spanæmia is sometimes induced which it may take months, nay years, to remove."

Another mode of endeavoring to apply statistical researches to our knowledge of therapeutics, is to compare cases treated by different methods as respects the duration of the disease. Is the duration of pneumonia, other things being equal, abridged by bloodletting? A fair exhibition of the results of Bouillaud's cases in this point of view, would be interesting, from the extent to which he pushes the detraction of blood. His published statistics, however, are rendered valueless, in this regard, by his peculiar system of reckoning the duration. He dates from the time the patient was admitted into the hospital, and considers convalescence established when the febrile movement is distinctly lessened, although it may not have ceased!† The investigations of Louis with reference to this point, are familiar to the members of the association. Analyzing two collections of cases, one embracing seventy-eight cases in which bloodletting was practiced, and the other twenty-nine cases in which this remedy was not employed, a comparison of the results appeared to warrant the conclusions, that bloodletting exerts a positive but limited influence in shortening the duration of the disease; that this influence is more marked in proportion as the bleeding is practiced near the date of the attack, and in cases in which the bleedings were copious and repeated, it is greater than in those in which a single bleeding only was resorted to, and a small quantity of blood taken; and, finally, that it does not succeed in arresting the disease.‡ These conclusions which, at the present moment, doubtless seem to many to offer too much encouragement to the

---

* Grisolle Pathologie Interne.

† Grisolle. Op. cit.

‡ Researches on Bloodletting. Translated by Dr. Putnam. Boston, 1836.

practitioner to employ bloodletting in pneumonia, at the time of their enunciation took the profession by surprise, on account of the moderate degree of confirmation which they afforded of the supposed importance of detracting blood in this disease. An analysis of a large number of recorded cases occurring at the Massachusetts General Hospital, by Prof. James Jackson, led to the same conclusion as respects abridgment of the disease,* the mean difference in duration between the cases treated with, and those without bloodletting, being about three days. More recently Grisolle has investigated this, in connection with other points relating to pneumonia, and he also arrives at results which appear to show a decided influence exerted by bloodletting in shortening the career of the disease. He is, however, strongly opposed to excessive bloodleting, and appears to establish by figures that its beneficial influence when moderately employed, is, to say the least, equal to that produced by the *coup-sur-coup* system of Bouillaud.

In view of the facts just referred to, with our present knowledge, it seems to be settled that in a series of cases the average duration of the disease under consideration, is rendered by bloodletting less than it would have been had the cases been treated without this measure. Whether the justness of this conclusion may be invalidated by continued and more extended researches, it is, of course, impossible to predict. The Vienna statistics of cases treated with gum water, and anodyne *tisanes*, give a mean duration of about twenty days. This is greater than the usual average duration in cases treated actively by different methods. It is not claimed that withholding active interference tends to shorten the duration of the disease; and it is to be borne in mind that in the Vienna collection were embraced cases of secondary pneumonia.

The tabulated cases of Dr. Ames, already referred to, give a mean duration (excluding children under five) of between eight and nine days. These cases were treated without either bleeding, antimony or mercury; the remedies employed being the tincture of aconite, sulphate of quinia and phosphorous. The duration in these cases is remarkably short, but how far this is due, on the one hand, negatively, to the non-employment of bleeding, antimony or mercury, severally or collectively; or, on the other hand, to the positive efficacy of the remedies substituted for those just named, is a problem, like many others pertaining to therapeutics, which it is difficult to resolve ration-

* Appendix to Putnam's translation of Louis on Bloodletting.

ally, and still more difficult by statistical demonstration. Dr. Ames' cases, at least, prove that bleeding is not essential to a brief duration. Farther statistical researches with respect to the point under consideration, are undoubtedly desirable. Data for prosecuting farther the inquiry may at the present moment be available, with which I am unacquainted, and which I am unable to seek for during the few days allotted to the preparation of this report.

Admitting the abridging influence of bloodletting, let us define a little more clearly the nature and extent of the admission. A series of cases treated with bloodletting give a shorter average duration than a series treated without bloodletting. This is very far from proving that in all, or in a majority of the cases embraced in the first series, the disease was shortened. It proves only that in a certain proportion this influence is produced. It does not prove that in some cases by the use of bloodletting the disease is not prolonged. It does not prove that the convalescence and recovery are more rapid and complete even in the cases in which the duration was abridged by bloodletting. In short, taking the deduction with respect to the influence of bloodletting in cases of this disease considered collectively, and applying it to cases as they occur individually in practice, in what manner does it dictate the propriety of resorting to this therapeutical measure? It dictates the employment of bloodletting when the circumstances in individual cases appear to indicate it, and not otherwise. The remarks here now lead me on to a subject which opens a wide field for discussion, viz., statistical results in their practical relations to the treatment of individual cases of disease. This subject I have considered somewhat in previous writings,* I cannot enter upon it here; and, indeed, I find myself in a train of investigation so broad in its scope, that to keep within the limits which are appropriate to a paper of this kind, I must abruptly stop, leaving what I have presented to suggest inquiries which, if followed out, would require, on my part, more time than I have at present at my command; and, on your part, perhaps, more patience than you would be willing to bestow.

In regard to the effect of bloodletting on the duration of pneumonia, the papers referred to the committee, communicated to the association by a member of the committee, contain certain facts which are to be noticed. Four cases of pneumonia are reported in which bloodletting was employed in all on the fourth day; in two of the cases being practiced three times, and in two once only. In one of the cases the patient was a child 4½ years of

* Clinical Reports on Continued Fever; and, Examination of the Numerical System in the New York Med. Journal.

age. The duration of the disease in these cases, respectively, are given as follows: 8, 5, 4, 2½ days. The mean duration is about 8¾ days. This is a small fraction below the mean duration in Dr. Ames' fifty-one cases treated with neither bleeding, antimony, nor mercury. The cases reported by a member of this committee, Dr. Burwell, were not given as illustrating a mode of practice to be pursued indiscriminately, or even generally, in the treatment of pneumonia; but as evidencing the success following bloodletting under circumstances which appeared to him to indicate in a special manner the employment of this measure. I shall not discuss the propriety of the practice pursued in these cases. Considering their termination and duration, the only question for discussion is, whether the detraction of blood was necessary; in other words, whether the favorable progress of the disease was not due to its natural tendencies irrespective of the treatment. This is a question more easily discussed than answered.

In connection, however, with the results in the cases reported by my associate on the committee, I will give the duration in six cases occurring during the present summer, and treated by me, with the exception of one case which I saw in consultation with Dr. Samo, but observed, and of which I took notes during the whole career of the disease. Five of these cases were male patients at the Hospital of the Sisters of Charity, and in the remaining single case the patient was a child, five years of age, treated in private practice. These are all the cases which I have observed during the present summer, in which bloodletting had not been practiced prior to their coming under my observation. In the case of the child a little antimony was prescribed. In this case the pneumonia was lobar, and uncomplicated with bronchitis. In all the other cases the treatment consisted of anodynes and stimulants. All the cases are fully recorded. In three of the cases an entire lung was consolidated. In the three remaining cases the inflammation was limited to the lower lobe. The cases came under observation at periods from the date of the attack, as follows: 5, 3, 8, 8, 6, 1 days. Reckoning from the initial chill, to a period when the pulse had fallen to the natural standard, or below it, (which not infrequently occurs) and the respirations but little, if at all accelerated, the duration in the cases respectively, was as follows: 11, 7, 14, 12, 7, 5. The mean duration is precisely 9⅓ days. The average duration exceeds that in the cases reported by Dr. Burwell, by a fraction less than a day. It is to be borne in mind that my cases were (with one exception) hospital cases; that the patients entered at a period from the date of attack, varying from three to eight days, had received no treatment prior to their admission, and in one-half the number of cases the inflammation extended over the entire lung on one side. In each of the cases the recovery was rapid. The duration to the time of being dressed, in the cases severally, is as follows: 14, 9, 20, 21, 8, 6 days. In three cases the patients remained in hospital for some time after being able to be discharged, two of them being employed as out-door assistants. The remaining two patients were discharged each on the fifteenth day from the date of the attack. Medical attendance on the child was discontinued on the eighth day.

The few cases reported by Dr. Burwell, and myself, thus present a mean duration not far from equal: the former treated with, and the latter without bleeding. In neither collection is the number of cases sufficiently large to warrant statistical deductions; but they serve, exhibited in contrast, to exemplify the general conclusion at which we must arrive after a consideration, be it ever so elaborate, of the evidence for or against the employment of bloodletting in pneumonia, viz., the propriety of this measure of therapeutics must be determined by the practitioner in each case separately, not by following any fixed formula; but by a rational interpretation of the indications which cases individually offer, guided by certain general principles regulating the employment of bloodletting in all acute inflammations. This conclusion embraces postulates, which to unfold and treat of fully would require more space than falls within the proper limits of this report. Assuming that we should be led to it had we time to follow the chain of considerations by which it is reached, diversity of practice among practitioners hinges mainly on the different views entertained respecting the general principles regulating the employment of bloodletting in all acute inflammations. Here, again, is a broad field of inquiry involved in a full investigation of the present subject. It will not, of course, be expected that I should enter upon it; but it *will* probably be expected that I shall not leave the subject without farther expression of my views respecting the treatment of pneumonia by bloodletting. My remarks already occupy so much space that I ought not to presume on your attention by extending much farther this report; and I shall, in conclusion, for the sake of brevity, embrace in a series of propositions certain practical points of consideration, which, if my leisure and your interest permitted, I should present in a shape not to be disposed of so cursorily. As it is, I can only add a very few brief comments, leaving a fuller exposition for oral discussion.

1. Primary pneumonia in the form in which it is usually presented in the adult, viz., confined to the lower lobe of one lung, tends so uniformly to recovery, that active interference by bloodletting, is not requisite as a means of securing a favorable issue of the disease. The objects, then, for which bloodletting is practiced in such cases must be embraced in the following enumeration: *a.* To arrest the disease. *b.* To abridge its duration. *c.* To limit the diffusion of the inflammation. *d.* To lessen the local morbid effects incident to pulmonary inflammation. *e.* To relieve pain and other distressing symptoms. *f.* To promote rapid convalescence and the complete restoration to health.

2. It appears to be fully established that bloodletting does not possess the power of arresting the disease. Its employment with a view to this object is therefore unwarrantable, and, if not otherwise indicated, must do harm in proportion to the want of ability, on the part of the patient, to bear up under the effects of so potent a therapeutical measure.

3. Clinical facts appear to show, that in a series of cases bloodletting exerts a limited influence in shortening the duration of pneumonia. We have not, however, evidence that this influence is exerted save in a certain proportion of cases, and it by no means authorizes the employment of bloodletting in cases indiscriminately. The effect on the duration of the disease is to be regarded as incidental to its judicious employment under other indications. To bleed every patient, because it has been found that in a large collection of cases the average duration is less in the cases bled than in those not bled, is a vicious application of statistical results. To abridge the duration of the disease, is, therefore, not a legitimate object for taking blood, disconnected from other objects.

4. The extension of inflammation from one lobe to another in the same side, or in the opposite side, which occurs in a certain proportion of cases, adds to the gravity of the disease, and it is, of course, desirable to prevent its occurrence. We have not, however, positive evidence, that bloodletting possesses any power to limit the diffusion of the inflammation. There is reason to believe that when the inflammation subsequently invades other lobes than the one primarily attacked, it is due to an internal tendency which, with our present knowledge and resources, we are unable to control. It is probable that instances in which an entire lung is affected, or in which the pneumonia is double, would be found not less numerous among a certain number of patients bled, than among the same number not bled. This is an interesting point to be settled by statistical researches.*

5. The local morbid changes incident to inflammation, viz., inflammatory

* Whether this point has been made the subject of statistical investigation, or whether data for such an investigation are available, the writer, at the moment of preparing this report, is unable to state.

exudation leading to obstruction of the air resides and interruption of the circulation through the parts inflamed, occur very uniformly in pneumonia, and, generally, early in the disease. It must be considered doubtful whether, to much extent, these changes may be lessened by bloodletting. The problem is a difficult one to determine, on the one hand, in cases in which bloodletting is practiced, whether the local changes are less than they would have been, had no blood been taken; and, on the other hand, in cases in which bloodletting is not practiced, whether the changes are greater than they would have been had this measure been employed. Considering that the physical signs of solidification are generally present in cases bled, as well as in those in which bloodletting is not practiced, the effect of the measure in limiting the amount of exudation is probably small, and exerted in only a certain proportion of cases. While we are not warranted in denying that bloodletting ever lessens the local changes in pneumonia, the grounds for expecting this result are not sufficient to constitute it an object for taking blood under circumstances when, if this effect be not produced, the condition of the patient will be damaged. It is obvious that with reference to this object any efficiency which bloodletting possesses depends on its employment within the first two, three or four days.

6. To relieve pain and other distressing symptoms, is a legitimate object of therapeutics, and that bloodletting in certain cases is followed by a marked abatement of the local symptoms cannot be doubted. In resorting to paliative measures, however, their ulterior effects are not to be overlooked. It would manifestly be bad practice to incur a risk of doing harm remotely, in order to secure a temporary alleviation of pain or distressing symptoms. Bloodletting, then, may be practiced early in pneumonia with a view to relieve the acute pain, and labored respiration present in some cases, but not under circumstances contra-indicating it, and not carried to an extent which will be likely to involve ulterior damage.

7. The objects to be attained by bloodletting, it is evident, relate to the existing disease. No one, it is to be presumed, advocates taking blood without reference to present indications or the immediate effects, but with a view to its influence on convalescence and the completeness of the recovery. On the other hand, the influence which the loss of blood may have in retarding convalescence, and leaving the system more enfeebled than it would have been from the effects of the disease alone, should restrain the practitioner from employing bloodletting in some instances, when, without any regard to its remote consequences, it might be indicated. The evils of unnecessary and excessive bloodletting in this disease are probably manifested by slow and incomplete restoration to health, rather than by an increased rate of mortality.

8. In view of the considerations contained in the preceding propositions, the objects for which bloodletting is practiced in pneumonia are few and limited; and in resorting to it the practitioner must be guided by circumstances pertaining to cases individually, and by the general principles regulating its application in acute inflammations. These circumstances and principles will supply the subjects of the few remaining propositions.

9. The circumstances indicating bloodletting in pneumonia, as the rule, belong only to an early period of the disease. Indications are rarely presented after solidification has occurred. The processes of restoration depending on the energies of the system are not likely to be favorably affected by the loss of blood; but, on the contrary, the recuperative power may be diminished.

10. General indications for bloodletting in acute inflammations, have relation to circumstances incidental to the inflammation rather than to the inflammation itself. They relate to the degree and character of the febrile movement; to the previous condition of the system; to the habits and constitution of the patient. Special indications in pneumonia relate to pain and embarrassment of respiration.

11. In pneumonia, as in acute inflammations generally, bloodletting is indicated, as the rule, only when symptomatic fever is present, and of a high grade, as denoted by increased frequency, and force of the pulse, the symptoms showing not simply morbid action, but augmented power of the circulation.

12. Polyæmia or plethora, previous good health, and vigor of constitution, are circumstances rendering bloodletting safe and proper; when, *per contra*, anæmia, feebleness of constitution, or impaired health from former disease, deprivations, over exertion, intemperance, etc., in connection with the same degree and extent of local inflammation, render it inappropriate and, perhaps, unsafe.

13. In conclusion, bloodletting in pneumonia, as in other acute inflammations, in some instances not only conduces to immediate relief, but abates, by an indirect action the intensity of the inflammation, and exerts a favorable influence on the progress of the disease. But in this, as in other affections, indiscriminately employed, or carried to an injudicious extent, it is a measure potent for harm, and may prove destructive. Employed in a manner to secure its benefits and avoid its evils, it is an useful remedy in a certain proportion of cases; and this proportion would be found to be not uniform in patients of different conditions; in the residents of a city and the country; in different countries and in different portions of the same country, and in successive years. Of cases occurring at the same time and in the same locality, in some it will be useful, in others hurtful. A just appreciation of the principles and rules which should regulate its employment, is essential; but in their application to individual cases, the line of practical conduct cannot be formularized, but must be left to the judgment, and, perhaps, it may be said the sagacity, which, in addition to scientific attainment, are necessary to form the skillful physician.

It will be observed that the remarks contained in the foregoing propositions have reference to primary, uncomplicated pneumonia in the adult. The applicability of bloodletting, as a general remark, is less when the disease is secondary, or complicated, or occurring at either of the extremes of life; but to waive consideration of the subject, in view of the length of this report, does not call for an apology.

"The diagnostic value of the buffy coat," on account of the space devoted to the other subjects referred to the committee, is reserved, with the consent of the association, for a separate report at some future day.

At the close of the reading of the foregoing, which was listened to with marked attention,

*Dr. Burwell* remarked that he had been much interested in this very able paper, and would suggest that in view of the lateness of the hour, and the probable publication of the proceedings, that any discussion should be deferred to another evening. He would say, however, that we should speak of pneumonia not as an abstraction, but as we find it. His colleague in ruling out pneumonia in children and the aged in discussing its mortality, had not come up to the scope of the resolution. We find pneumonia in these classes, and are obliged to treat it, and though adults usually were but little endangered by pneumonia, it is among children that we find the largest number of cases and the greatest benefit from venesection. The responsibility of the practitioner is as great for sins of omission as for those of commission. He objected entirely to the statistical comparison of cases bled or not bled. His own cases furnished such a comparison — cases which not being bled did equally well with those bled; but that did not impair the value of venesection, and the individual judgment of the practitioner was the only guide to decide the question. As illustrating why we should not compare cases bled or not bled, he would mention a case reported in the July Journal: a boy of 18 years, whom he had bled once, and whom he had expected to bleed on the succeeding day, and, whether wisely or foolishly, it was not for him to say, he had promised a rapid cure and convalescence. Before the time for the expected bleeding, the patient was sent to the hospital; he was not bled the second time, but after lingering there for three weeks, confined to his bed, he left the house and came to him again in a feeble condition, and was two months before he regained his health. Now here was a case, which, had it been bled again, he had no hesitation in saying would have had a brief and rapid convalescence. Such a case showed the impropriety of a comparison of cases bled with those not bled.

*Dr. Flint* explained, that in presenting his report he did not wish to evade, but hoped for discussion. Undoubtedly the cases at the two extremes of life required consideration; he did not waive them because he thought them unimportant, but the discussion was limited, and the subject before them was pneumonia itself, not pneumonia associated with, and modified by, other conditions, which were accidental and not intrinsic to the disease. It was this view that gave interest to the fact that adult cases terminated favorably, while the very young or old ended fatally. His colleague had said that the success of cases not bled should not be compared with that of those bled, inasmuch as the first might not have needed the lancet. Did he not in this concede the question that the intrinsic nature of the disease was toward recovery? Would he only admit statistical proof in favor of bloodletting, and deny it admission when the lancet was omitted? He had not intended to discúss the treatment of individual cases. But Dr. Burwell had instanced a case which, in his (Burwell's) opinion, should have been bled a second time. This patient came into his (Flint's) hands at the hospital, in a condition, which in his individual judgment, did not call for the lancet. In preparing his report he had designedly omitted any mention of this case, because he thought that no fair conclusions could be drawn from it. Dr. B. assumes that another bleeding would have shortened the convalescence. But here were other cases in the same ward, which were not depleted, which had rapid and safe recoveries. If any conclusion at all should be drawn from this case (which he freely admitted there should not) it would be inevitably that the bloodletting prolonged the disease. The case was as good for him as for his colleague.

The reports of the committee were then accepted, and ordered for publication.

*Dr. Rochester* stated in explanation, that at the previous discussion of this subject, he had spoken of thirteen cases in the hands of a friend, none of which had been bled. He should have said that none of them had been bled by his friend, and six of them not at all, but that seven of them had been depleted either generally or locally, before coming under the treatment of the gentleman alluded to.

*Dr. Burwell* explained, that in all those cases which he had reported as having been bled on the fourth day, the venesection was performed at the close of the day. It would, therefore, be more proper to say that the blood was taken on the fifth day.

*Dr. Lay* then read a brief report of a case presenting anomalous symptoms, which is omitted here, as it may again come up for consideration.

*Dr. Baker* moved that a committee of three be appointed to revise the by-laws.

*Dr. Newman* objected, inasmuch as the association might conclude to apply for a charter, and a second revision would then be necessary.

*Dr. Wilcox* moved to amend, by making it the duty of the committee to take into consideration the subject of a legal organization. The amendment was accepted, and the motion passed. The chair appointed Drs. Baker, Wilcox, and Newman, such committee, and the association adjourned.

SANFORD B. HUNT, Secretary.

